# Electroacupuncture at ST36 Ameliorates Gastric Emptying and Rescues Networks of Interstitial Cells of Cajal in the Stomach of Diabetic Rats

**DOI:** 10.1371/journal.pone.0083904

**Published:** 2013-12-31

**Authors:** Yan Chen, Juan Juan Xu, Shi Liu, Xiao Hua Hou

**Affiliations:** Division of Gastroenterology, Union Hospital, Tongji Medical College, Huazhong University of Science and Technology, Wuhan, China; University of California, Los Angeles, United States of America

## Abstract

Depletion of interstitial cells of Cajal (ICC) is certified in the stomach of diabetic patients. Though electroacupuncture (EA) at ST36 is an effective therapy to regulate gastric motility, the mechanisms of EA at ST36 on gastric emptying and networks of ICC remain to be elucidated. The aims of this study were to investigate the effects of EA on gastric emptying and on the alterations of ICC networks. Rats were randomized into the control, diabetic rats (DM), diabetic rats with sham EA (DM+SEA), diabetic rats with low frequency EA (DM+LEA) and diabetic rats with high frequency EA groups (DM+HEA). The expression of c-kit in each layer of gastric wall was assessed by western blotting. The proliferation of ICC was identified by immunolabeling of c-kit and Ki67 as the apoptosis of ICC was examined by TUNEL staining. The results were as follows: (1) Gastric emptying was severely delayed in the DM group, but accelerated in the LEA and HEA group, especially in the LEA group. (2) The expression of c-kit in each layer was reduced apparently in the DM group, but also up-regulated in the LEA and HEA group. (3) Plentiful proliferated ICC (c-kit+/Ki67+) forming bushy networks with c-kit+ cells were observed in the LEA and HEA group, while the apoptotic cells (c-kit+/TUNEL+) were hardly captured in the LEA and HEA group. Collectively, low and high frequency EA at ST36 rescue the damaged networks of ICC by inhibiting the apoptosis and enhancing the proliferation in the stomach of diabetic rats, resulting in an improved gastric emptying.

## Introduction

Kinds of gastrointestinal motility disorders ranging from functional dyspepsia to gastroparesis severely affect the quality of life of patients with decades of diabetes mellitus [1]. Gastroparesis, characterized by delayed gastric emptying, occurs up to 30% of individuals with Type 1 diabetes [2]. Although various therapeutic interventions including pharmaceutical treatment and some non-drug treatments have been tried, the curative effects for gastroparesis remain unsatisfactory.

Acupuncture has been empirically used to treat gastrointestinal disorders in oriental countries for thousands of years. Electroacupuncture (EA), which is a modification of traditional acupuncture, is more suitable for clinical and basic research settings for its reproducibility. *Zusanli* (ST36) is a common acupoint frequently used to treat gastrointestinal diseases. In recent years, the effects of EA at ST36 on gastric motility have been evaluated in numerous studies. For animals, EA at ST36 significantly increased gastric motility in conscious rats and improved delayed gastric emptying in STZ-induced diabetic rats [3], [4]. Moreover, in humans, EA at ST36 accelerated gastric emptying and relieved dyspeptic symptoms in functional dyspepsia patients and diabetic individuals with gastroparesis [5], [6]. Although in these studies only low frequency EA was adopted, high frequency EA was also reported to promote esophageal and distal colon motility in animals [7], [8]. Thus, the effects of different frequency EA on gastric motility and the underlying mechanisms draw people's attention.

Interstitial cells of Cajal (ICC), well-established as pacemakers generating and propagating slow waves, have gained significance in regulating gastrointestinal motility over several decades [9]. Three subtypes of ICC including intramuscular ICC (ICC-IM), myenteric ICC (ICC-MY) and submucosal ICC (ICC-SM) form intact networks in the gastric wall [10]. Networks of ICC are not static even in physiological conditions; apoptosis and transdifferentiation lead to ICC loss, while ICC also can be restored by proliferation, replenishment from stem cells and increasing survival [11], [12]. Extensive studies have documented that loss and damage of ICC caused disordered gastrointestinal motility in animal models and patients with diabetes [13]–[15]. Recently, further studies reported that EA facilitated the increase of colonic ICC in slow transit constipation rats and rats after enteroenterostomy [16], [17]. Our recent study also demonstrated that EA at ST36 could restore impaired ICC in the colon of diabetic rats [18]. Meanwhile, it is concerned that EA promoted proliferation and inhibited apoptosis of brain cell in the hippocampus [19], [20]. However, there is a blank about the effects of EA at ST36 on ICC in diabetic gastroparesis and the mechanism whether the proliferation and apoptosis of ICC are involved.

Therefore, the aims of this study were to: 1) evaluate the effects of different frequency EA on gastric emptying; 2) investigate the effects of EA at ST36 on ICC networks in diabetic rats; 3) certify whether the apoptosis and proliferation of ICC were involved.

## Materials and Methods

### Ethics statement

The rats received human care and this study was carried out strictly in accordance with the recommendations in the Guide for the Care and Use of Laboratory Animals of the National Institutes of Health, with all operations approved by the ethical guidelines from the Animal Care and Use Committee of Tongji medical college, Huazhong University of Science and Technology.

### Animals

Male Sprague-Dawley rats weighed 250–300 g were selected in the present study. Rats were obtained from the Experiment Animal Center of Tongji Medical College (Huazhong University of Science and Technology, Wuhan, China) and kept under normal laboratory conditions (22°C, 12/12 h light-dark cycle) with free access to food and sterile water. After one week of adaptive feeding, animals were officially adopted into our experiments.

### Diabetic model

After overnight fasting but free access to water, the models were established with a single intraperitoneal injection of streptozotocin in rats (STZ, 60 mg/kg, i.e. 1 ml/100 g body weight; Alexis Biochemicals, San Diego, CA, USA), freshly dissolved in 20 mM citrate buffer solution (pH 4.5; Sigma, St Louis, MO, USA). The control group was injected with an equal volume of citrate buffer. All rats were housed in the same condition, with water deprivation for 4 h but *ad libitum* feeding. One week after the injection, diabetes was confirmed in different time by measuring glucose in a drop of whole blood obtained by puncturing the tip of tail vessel. A blood glucose level of ≥16.7 mmol/L was taken as a standard for successful model. Blood glucose was screened punctually before the injection and 1st, 4th and 8th week after the injection. Furthermore, weight loss, polydipsia and polyuria were further indicators to evidence the successful inducement of diabetic rat.

### Experimental protocols

Rats were randomized into five groups (10 rats each group), including the control, diabetic group (DM), diabetic rats with sham EA group (SEA, only acupuncture without electric current, 30 min/d), diabetic rats with low frequency EA group (LEA, 10 Hz, 1–3 mA, 30 min/d) and diabetic rats with high frequency EA group (HEA, 100 Hz, 1–3 mA, 30 min/d). EA was implemented at acupoint ST36 with an electrical stimulator (G6805-2A; Shanghai Huayi Medical Instrument Factory, Shanghai, China) everyday for 8 weeks. The electric current was established according to slightly trembling of the lower limbs. To achieve the purpose of eliminating the influence of stress, animals were allowed to move freely in their own casing during the EA procedure.

After successive intervention for 8 weeks, the rats in each group were tested for gastric emptying. Then they were sacrificed, and specimens of antrum were carefully obtained. Each specimen was divided into several pieces, a relatively small one was stored at −80°C for western blotting analysis after layers freshly detached and a piece was placed into 2.5% glutaraldehyde for electron microscopy, and the rest were fixed in Zamboni's fixative (2% paraformaldehyde and 1.5% saturated picric acid solution in 0.1 M phosphate buffer solution (PBS, PH 7.4) for immunofluorescence staining.

### Measurement of gastric emptying

Gastric emptying was performed by a modification as described previously [21]. The test meal containing phenol red (0.5 mg/ml) as indicator and carboxymethylcellulose (15 mg/ml) was constantly stirred and then held at 37°C. After food-deprived overnight, rats were received 2 ml phenol red meal administered via a gavage needle. Thirty minutes later, animals were rapidly sacrificed by cervical dislocation. The whole stomach was removed carefully after ligation at the cardia and pyloric, and then the stomach was open and its contents were poured into a test tube and washed with 40 ml distilled water. At the end of the experiment, NaOH solution (20 ml, 1 M) was added to each tube to develop the maximum intensity of color. The absorbance of the sample read at 560 nm with a spectrophotometer (HITACHI, U-2900) was to reflect the amount of phenol red remaining in the stomach. The rate of gastric emptying was calculated according to the following formula: Gastric emptying (%) = 100×(1 − X/Y), X represents absorbance of phenol red collected from the stomach of animals sacrificed 30 min after the test meal. Y represents absorbance of phenol red recovered from the stomach of control animal killed immediately after the administration of the test meal.

### Western Blotting

Fresh samples of gastric antrum were pinned to a Sylgard dish and each layer was detached carefully by sharp dissection after removal of the mucosa. With the help of fine-pointed forceps and microsurgical scissors under a dissecting microscope, layers of ICC-SM, ICC-IM and ICC-MY were obtained. All the manipulations were done on the ice to avoid the protein loss. And then samples of ICC-SM, ICC-IM and ICC-MY were stored at −80°C.

Fresh-frozen samples were crushed into the cell suspension and homogenized in RIPA Buffer (Upstate, Temecula, CA, USA) with protease inhibitor (Sigma Chemical Co., St Louis, MO, USA). The mixture was centrifuged at 12000 g for 10 min at 4°C, and the supernatants were collected as the total protein. The protein concentration was determined by the Bradford method. Subsequently, equivalents of 50 mg of extracted proteins were gradually separated by 10% sodium dodecylsulfate polyacrylamide gel electrophoresis (SDS–PAGE) and transferred to PVDF membrane (Millipore, Bedford, MA, USA). Followed blocking nonspecific binding spots with 5% nonfat dry milk in Tris-buffered saline containing 0.1% Tween 20 (TBST) at room temperature for 1 h, these membranes were incubated with primary antibody to anti-c-kit (1∶200, Santa Cruz Biotechnology, Inc) overnight at 4°C along with rabbit anti-mouse Actin (1∶400, Santa Cruz Biotechnology, Inc) served as the internal control. After three washes with TBST, the membranes incubated with HRP-linked secondary antibody (HRP-linked rabbit anti-goat and HRP-linked goat anti-mouse, 1∶5000) for 1 h at room temperature. Followed with three washes of TBST, the bands were visualized by chemical reaction with enhanced chemiluminescence agent (Amersham Pharmacia Biotech, Piscataway, NJ) and the blot was subjected to autoradiography. Densitometry analysis was performed by Quantity One software (Bio-Rad Technical Service Department, Version 4.6.2).

### Immunofluorescence studies

For whole-mount staining preparations, the stomach was opened and rinsed several times with PBS. Gastric antrum was pinned to a Sylgard dish and stretched to 150% of their original size, and then fixed with Zamboni's fixative for 24 h at room temperature. The fixed tissues were washed with PBS and then the mucosa was removed by sharp dissection. ICC-SM locating at the submucosal surface of the circular muscle layer, ICC-IM intermingling among smooth muscle cells and ICC-MY together with myenteric nerve plexus were carefully prepared using fine-pointed forceps and microsurgical scissors under a dissecting microscope.

The immunostaining procedures for detecting ICC networks and proliferation have been previously described [22]. Ki67, a nuclear protein expressed during all the active phases of cell and vanished in the resting cell, has been applied to detect the proliferative ICC [23]. After washing six times with PBS for 1 h, tissues were incubated with 5% normal bovine serum in PBS containing 0.3% Triton X-100 (PBST) for 1 h at room temperature and then incubated with primary antibody c-kit (1∶100; Santa Cruz, CA, USA) and Ki67 (1∶100; Abcam, Cambridge, UK) overnight at 4°C. After rinsing in PBST for 30 min, the immunoreactivity was detected using Alexa Fluor 594-conjugated secondary antibody (donkey anti-goat IgG, 1∶100) and Alexa Fluor 488-conjugated secondary antibody (donkey anti-rabit IgG, 1∶100) at 37°C for 1 h, followed by rinsing in PBS three times for 30 min, and incubated with DAPI (0.1 mg/ml; Sigma, St Louis, MO, USA) for 30 min as a nuclear counter stain. Rinsed in PBS twice, tissues were unfolded on a slide. A confocal laser scanning microscope (Nikon, Japan) was used to examine the immunolabeled tissues. In order to include all the levels of positively stained cells and processes, the optimal Z stacking of confocal images was set at 5 µm intervals.

Apoptosis of ICC were detected with TUNEL labeling, an apoptosis detection Kit (Roche, Germany). Whole-mount preparations were incubated with primary antibody c-kit (1∶100; Santa Cruz, CA, USA) overnight at 4°C and secondary antibody (Alexa Fluor 594 donkey anti-goat IgG, 1∶100) at 37°C for 30 min. Under a humidified and dark circumstance, the tissues were incubated with TUNEL reaction buffer (labeling solution: enzyme solution = 9∶1) at 37°C for 1 h. After washed three times for 30 min to remove unconnected fluorescein-dUTP and incubated with DAPI as a nuclear counter stain, the specimens were observed under a confocal laser scanning microscope.

### Electron microscopy

After the mucosa removed, specimens of antrum were immersed in a fixative solution of 2.5% glutaraldehyde at 4°C for 2 h. Then, they were rinsed with 0.1 M PBS twice for 20 min, post-fixed in 1% OsO_4_ (pH 7.4) for 1 h, dehydrated with graded alcohol, clarified in propylene oxide and embedded in Epon using flat moulds. Ultrathin sections were obtained with an ultramicrotome (Leica ULTRACUTU, Germany), and stained with lead citrate for 10 min before viewing with a transmission electron microscope (Tecnai G^2^12, FEI, Netherlands).

### Statistical analysis

For immunofluorescence staining analysis, gastric antrum from each experimental animal was sampled in the same location of the stomach. ICC was recognized by c-kit immunoreactivity and the presence of abundant cellular processes. Proliferating and apoptotic ICC were identified by c-kit immunoreactivity and Ki67/TUNEL stained nucleus enclosing with c-kit positive membrane structures. Ten random fields (×200 magnification, 0.2607 mm^2^) per whole-mount preparation were taken for photographs of c-kit positive cells, c-kit+/Ki67+ and c-kit+/TUNEL+ labeled cells. These photographs were also employed to evaluate the thickness of the layers in each stack with the help of DAPI labeled nuclei as a marker of the upper and lower limits of the layers. The numbers of c-kit positive cells, c-kit/Ki67 and c-kit/TUNEL double labeled cells were counted with Image-ProPlus 5.0 (Media Cybernetics). All data were expressed as mean ± SEM. At least ten visions were taken in each animal. The analysis of variance (ANOVA) and the Student's t-test were used to compare the differences among different groups. P value less than 0.05 was taken as statistically significant difference.

## Results

### Body weight and blood glucose level

After 8 weeks, accidental death occurred in the DM group (two rats) and in the SEA group (one rat). The rats were screened for the same baseline body weight among the groups (Table 1). At the end of 1, 4 and 8 weeks, body weight of the DM group was significantly decreased compared with the control (*P* = 0.009, 0.000 and 0.000). There was no significant difference between the SEA and DM group (*P*>0.05). However, in contrast to the DM group, the body weight was elevated in the LEA group at 8 weeks (*P*<0.001) and HEA group at 4 and 8 weeks (*P* = 0.003, *P*<0.001 separately).

**Table 1:** Body weight measured during the experiment in different groups.

Body weight (g)	Control	DM	DM+SEA	DM+LEA	DM+HEA
0 week	275.00±5.00	278.25±5.02	280.00±5.53	277.50±3.27	282.00±4.16
1st week	318.13±4.72	263.25±10.85*	274.90±4.98	261.67±8.08	277.90±4.16
4th week	362.50±6.54	248.75±7.75*	252.22±7.41	291.10±15.88	311.30±10.93#
8th week	421.00±11.19	228.13±6.33*	250.33±14.43	327.00±8.88#	348.80±15.44#

Values were expressed as mean ± SEM, N = 47 (n = 10, 8, 9, 10, 10, respectively).

*P<0.05 compared with the control,

^#^ *P*<0.05 compared with the DM group in the same time point.

As shown in Table 2, no differences were noted in the baseline blood glucose level among the groups. The diabetic models were established successfully with the high blood glucose level in the DM group compared with the control at the end of 1, 4 and 8 weeks (all *P*<0.001). Meanwhile, the blood glucose levels in the SEA, LEA and HEA groups were not changed significantly compared with the DM group (all *P*>0.05).

**Table 2:** Blood glucose measured during the experiment in different groups.

Blood glucose (mmol/L)	Control	DM	DM+SEA	DM+LEA	DM+HEA
0 week	6.23±0.38	6.46±0.51	6.31±0.43	6.46±0.37	6.36±0.57
1st week	6.66±0.66	28.41±1.34*	28.89±0.96	29.09±1.26	27.99±1.30
4th week	6.80±0.57	31.61±0.68*	29.83±1.47	27.75±1.68	25.96±1.91
8th week	5.81±0.36	30.93±1.02*	28.84±1.55	28.29±1.67	28.79±1.04

Values were expressed as mean ± SEM, N = 47 (n = 10, 8, 9, 10, 10, respectively).

*P<0.05 compared with the control in the same time point.

### Effects of EA on gastric emptying

**Figure 1:**
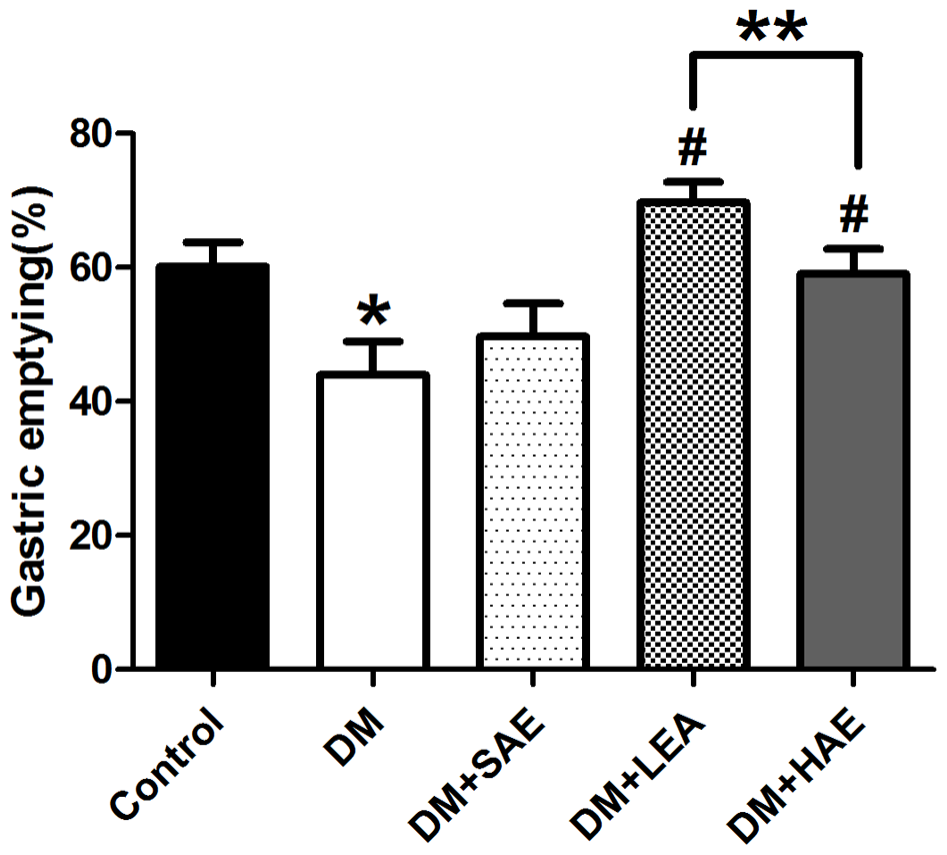
Effects of EA at ST36 on gastric emptying. Gastric emptying was dramatically delayed in the DM group (n = 8) compared with the control (n = 10, *P* = 0.012). Meanwhile, LEA and HEA significantly promoted the delayed gastric emptying (n = 10, *P* = 0.000; n = 10, *P* = 0.013). Significant difference was also found between the LEA and HEA group (*P* = 0.04). *P<0.05 compared with the control, ^#^ *P*<0.05 compared with the DM group, **P<0.05 comparisons between the LEA and HEA group.

Fig.1 showed the effects of EA at ST36 on gastric emptying in different groups. The gastric emptying of the DM group was dramatically delayed in contrast to the control (*P* = 0.012). Compared with the DM group, SEA had no significant effect on the gastric emptying (*P* = 0.338), but LEA and HEA markedly accelerated the delayed gastric emptying from 43.96±5.02% to 69.72±3.02% and to 59.06±3.70% (*P*<0.001 and *P* = 0.013, respectively). Comparison between the LEA and HEA group, statistical significance was achieved (*P* = 0.04), representing that the effect of LEA obviously exceeded that of HEA.

### Effects of EA on the expression of c-kit

**Figure 2:**
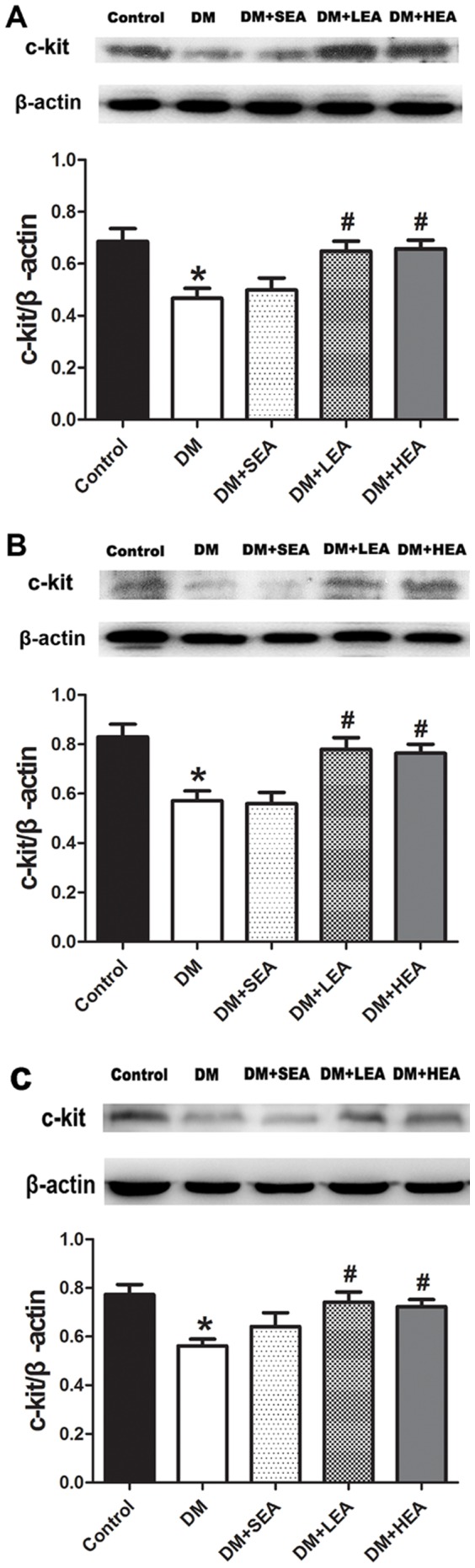
Expression of c-kit protein in ICC-IM (A), ICC-MY (B) and ICC-SM (C) layer. Compared with the control, the expression of c-kit in each layer was decreased in the DM group. However, it was markedly increased in the LEA and HEA group. *P<0.05 compared with the control, ^#^ *P*<0.05 compared with the DM group.

From Fig.2, the expression of c-kit in each layer of gastric wall was assessed. In ICC-IM, c-kit in the DM group was obviously decreased compared with the control (0.47±0.04 vs 0.69±0.05, *P* = 0.001). Conversely, LEA and HEA dramatically enhanced the expression of c-kit compared with the DM group (0.65±0.04 vs 0.47±0.04, *P* = 0.004; 0.66±0.03 vs 0.47±0.04, *P* = 0.002, respectively). In ICC-MY, the expression of c-kit was significantly reduced in the DM group in contrast to the control (0.57±0.04 vs 0.83±0.05, *P*<0.001). But significant difference was both achieved in the LEA and HEA group compared with the DM group (0.78±0.05 vs 0.57±0.04, *P* = 0.002; 0.76±0.04 & 0.57±0.04, *P* = 0.004, respectively). In ICC-SM, the c-kit of the DM group was evidently lessened on the basis of normal level (0.56±0.03 vs 0.77±0.04, *P* = 0.001). Consistently, LEA and HEA obviously increased the expression of c-kit compared with the DM group (*P* = 0.003 and *P* = 0.007). However, no significant difference was found between the SEA and DM group in ICC-IM, ICC-MY and ICC-SM (*P* = 0.597, *P* = 0.853 and *P* = 0.172).

### Effects of EA on ICC networks

**Figure 3:**
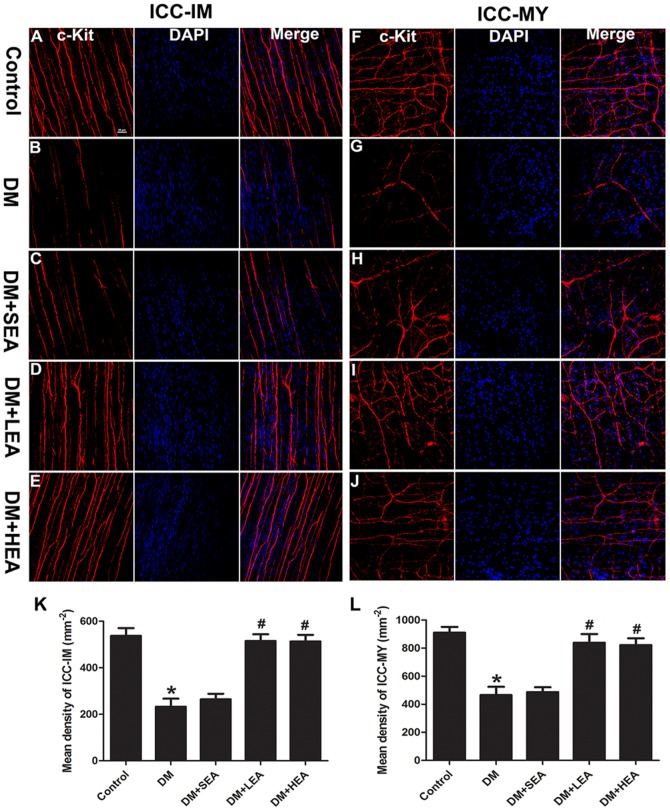
Confocal micrographs of ICC-IM (A–E) and ICC-MY (F–J) labeled with c-kit (red) and DAPI (blue). Numerous ICC with long and plenty branches were observed in the control group. Networks of ICC-IM and ICC-MY were incomplete with fractured processes in the DM group. Similarly, these alterations of ICC networks were also noted in the SEA group. However, in the LEA and HEA group, ICC-IM and ICC-MY projected elongated processes to form complicated cellar networks. Quantitative analysis of ICC-IM and ICC-MY were preformed in different groups in **K** and **L**. *P<0.05 compared with the control, ^#^ *P*<0.05 compared with the DM group. Scale bars  = 50 µm.

Alternations of ICC networks especially ICC morphology and density were displayed in Fig.3. There was no significant difference of the layers thickness among the groups. Associated with smooth muscle cells, c-kit immunoreactivity for ICC-IM was observed in the muscle layers, showing that ICC-IM had two polar processes forming a cellular network in the control group. In the DM group, the long processes of ICC-IM was impaired apparently and the density of c-kit positive cell was reduced to about 50% of normal control (*P*<0.001). Compared with the DM group, no significant change was noted in the SEA group. However, an almost intact cellular network was visualized in the LEA and HEA group and the density of c-kit positive ICC-IM nearly restored to the normal level (both *P*<0.001, compared with the DM group).

ICC-MY winding around the myenteric plexus were located between longitudinal and circular smooth muscle layers. Multipolar ICC-MY with numerous branches forming an intact cellular network were clearly showed in the control group. In the DM group, c-kit+ cells appeared with slender cell bodies and fractured processes, and the density of ICC-MY was obviously decreased (*P*<0.001). In the SEA group, the density of ICC-MY were not significantly altered compared with the DM group (*P* = 0.762). However, an almost intact cellular network existed around the myenteric plexus and the ICC-MY density partially recovered in the LEA and HEA group (both *P*<0.001, compared with the DM group).

### Effects of EA on apoptosis of ICC

**Figure 4:**
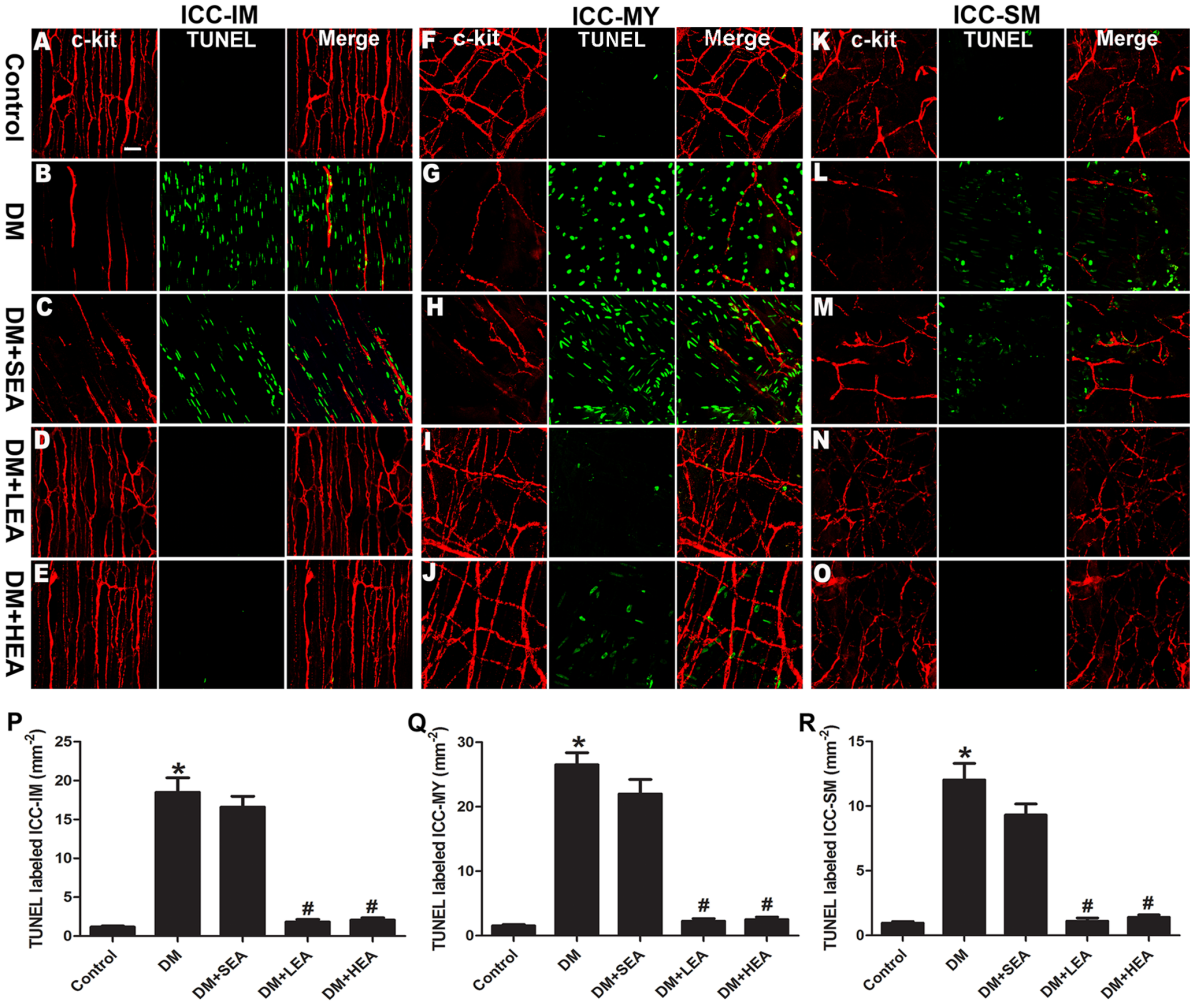
Confocal micrographs of apoptotic ICC-IM (A–E), ICC-MY (F–J) and ICC-SM (K–O). Only a few number of c-kit+/TUNEL+ cells were found in the control group (**A**, **F**, **K**), while the damaged networks of ICC in the DM group were flood by TUNEL+ nucleus (**B**, **G**, **L**). It was also revealed that plenty of apoptotic ICC-IM (**C**), ICC-MY (**H**) and ICC-SM (**M**) in the SEA group. The cellular networks of all layers in the LEA (**D**, **I**, **N**) and HEA group (**E**, **J**, **O**) presented with few apoptotic ICC. Quantitative analysis of ICC-IM, ICC-MY and ICC-SM were carried out in different groups in **P-R**. *P<0.05 compared with the control, ^#^ *P*<0.05 compared with the DM group. Scale bars  = 20 µm.

Apoptosis of ICC was detected by TUNEL labeling to determine whether apoptosis was involved in deficiency of ICC in diabetes (Fig.4). From whole-mount preparations of the control, few nuclei of ICC were stained by TUNEL method in muscle layer, myenteric layer and submucosa layer. Plenty of c-kit+/TUNEL+ ICC-IM, ICC-MY and ICC-SM were observed in the DM group (18.48±1.88 mm^−2^, 26.51±1.87 mm^−2^ and 12.03±1.27 mm^−2^, respectively). The c-kit+ cellular networks were incomplete and the apoptotic ICC presented as shortened processes with fewer branches. Large numbers of c-kit+/TUNEL+ cells were also flood in all layer of the SEA group. Under the implementation of LEA and HEA, apoptotic kit+ cells were hardly found in ICC-IM (1.79±0.33 mm^−2^ and 2.05±0.30 mm^−2^), ICC-MY (2.24±0.40 mm^−2^ and 2.48±0.40 mm^−2^) and ICC-SM (1.09±0.25 mm^−2^ and 1.39±0.20 mm^−2^). Cellular networks revealed by c-kit immunostaining showed high density and thickness of cellular processes in the LEA and HEA group.

### Effects of EA on proliferation of ICC

**Figure 5:**
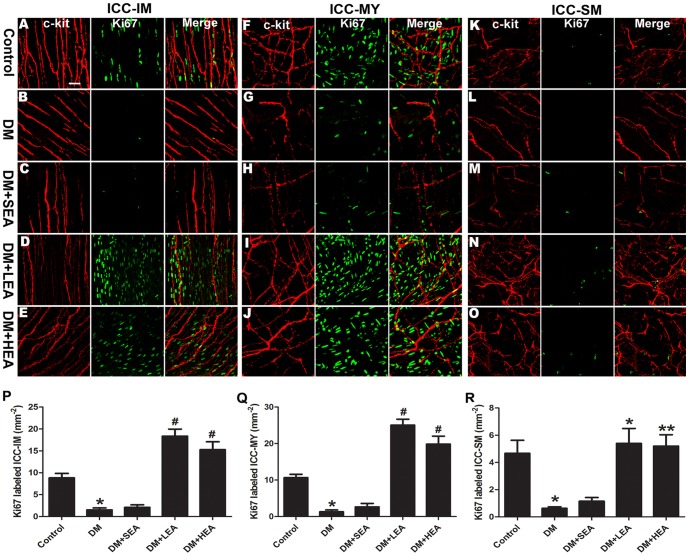
Confocal images of ICC labeled with c-kit (red) and Ki67 (green). Compared with the control group (**A**, **F**, **K**), proliferating ICC were hardly observed in ICC-IM (**B**), ICC-MY (**H**) and ICC-SM (**M**) of the DM group. Few of c-kit/Ki67 double labeled cells were recognized in the SEA group (**C**, **H**, **M**). Under the effects of LEA and HEA, abundant proliferating ICC forming intact networks were observed in ICC-IM (**D**, **E**), ICC-MY (**I**, **J**) and ICC-SM (**N**, **O**). Quantifications of ICC-IM (**P**), ICC-MY (**Q**) and ICC-SM (**R**) were executed in different groups. *P<0.05 compared with the control, ^#^ *P*<0.05 compared with the DM group. Scale bars  = 20 µm.

Considering the increased number of ICC in the LEA and HEA group, Ki67-positive ICC was detected to reveal the proliferation of ICC in the three layers (Fig.5). In the control group, a number of c-kit/Ki67 double labeled cells were observed in ICC-IM (8.82±1.02 mm^−2^), ICC-MY (10.64±0.92 mm^−2^) and ICC-SM (4.67±0.96 mm^−2^). The number of ICC with fractured branches was remarkably reduced and the networks were very sparse with few proliferating cells both in the DM and SEA group. However, in the LEA and HEA group, c-kit/Ki67 double labeled cells in ICC-IM were characterized by closely adjacent cell bodies and relatively bipolar processes and the mean density reached to 18.37±1.60 mm^−2^ and 15.27±1.81 mm^−2^ (both *P*<0.001). Similarly, abundant c-kit+/Ki67+ ICC-MY in the LEA and HEA group (both *P*<0.001) forming intact networks with c-kit+ cells performed a round cell body and long slender processes. In ICC-SM, accompanied by an intact network of ICC, the density of proliferating cells in the LEA and HEA group were relatively higher in contrast to the DM group (*P* = 0.016 and *P* = 0.003).

### Effects of EA on ultrastructural alterations of ICC

**Figure 6:**
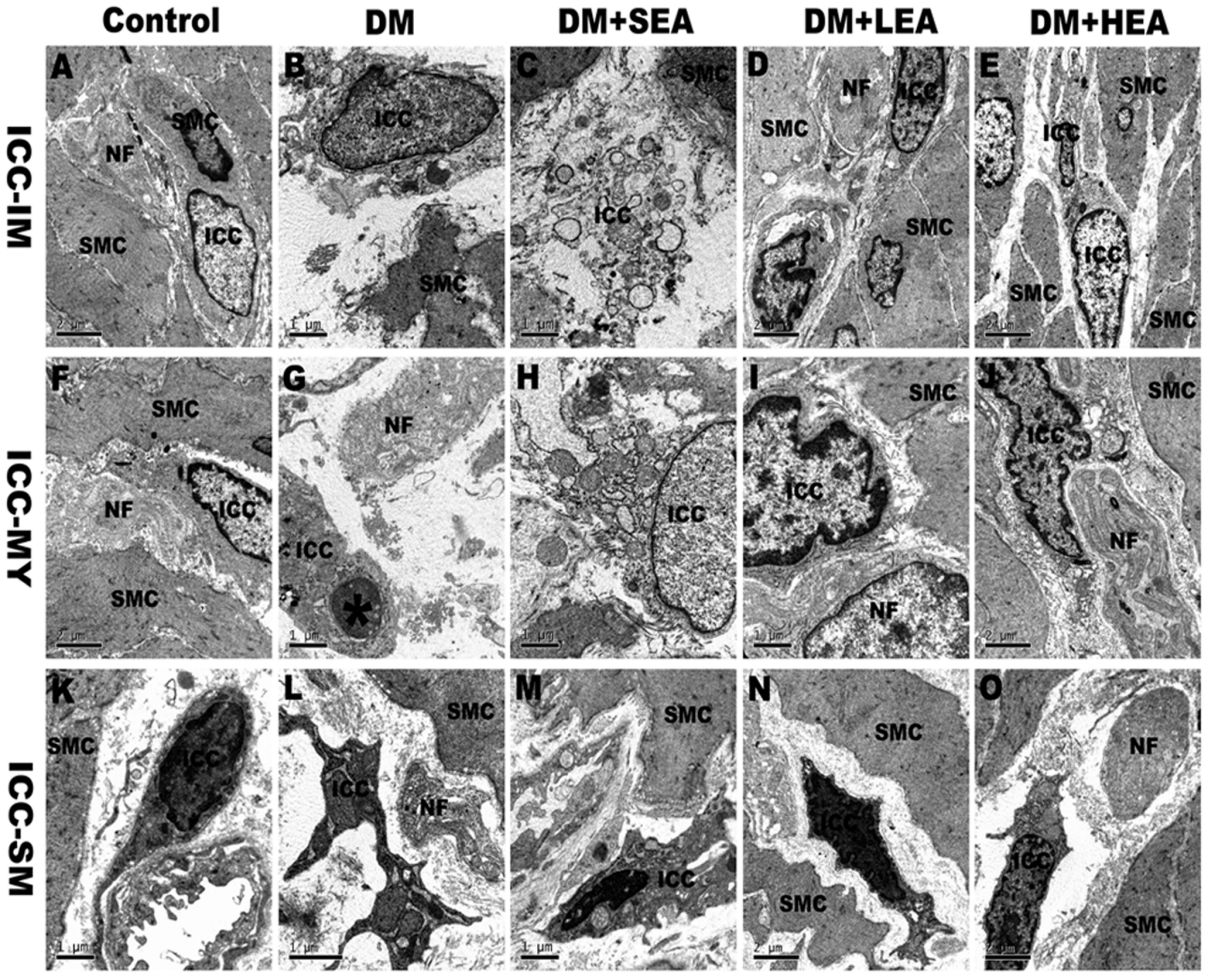
Ultrastructural changes of ICC in gastric antrum. ICC-IM and ICC-MY were observed to connect closely with nerve fibers (NF) and smooth muscle cells (SMC), with synapse-like structures formed between processes of ICC and nerve fibers in the control (**A**, **F**). In the DM group, serious injured ICC, with swollen mitochondria and endoplasmic reticulum and fractured branches, were interposed between degenerated NF and incomplete SMC, resulting in wide gaps (**B**, **G**). An apoptotic nuclei (*) was observed in the ICC. In the SEA group, swollen ICC was seen with incomplete membrane (**C**, **H**). However, in the LEA and HEA group, ICC was organelle-rich and integrated with neighboring NF and SMC (**D**, **E**, **I**, **J**). ICC-SM were present with polymorphic dense bodies (**K–O**). Swollen mitochondria with disrupted cristae were found in the DM and SEA group (**L**, **M**). Scale bars are as indicated in each panel.

Typical ultrastructural characteristics of ICC were revealed by transmission electron microscopy (Fig.6). In the control group, ICC-IM and ICC-MY rich of well-developed cell organelles extended long processes containing mitochondria, and rough and smooth endoplasmic reticulum. Polymorphic ICC-SM located at the submucosal surface of circular muscle layer showed higher electron-dense cytoplasm, numerous mitochondria and endoplasmic reticulum. In the DM group, a dramatical decrease in the number of ICC with damaged ultrastructural features was examined by electron microscopy. These cells with swollen mitochondria, disrupted cristaed and condensed chromatin showed incomplete membrane and shorten processes. These changes were also seen in the SEA group. In the LEA and HEA group, the number of ICC was significant increased and they connected closely with nerve fibers and smooth muscle cells forming synaptic structure and gap junctions. Clear mitochondria crest, orderly distributed rough endoplasmic reticulum and ribosomal particles were considered to be classical features of ICC-IM, IM-MY and ICC-SM.

## Discussion

In the present study, we found that both low and high frequency EA at ST36 suppressed the apoptosis of ICC and activated the proliferation of ICC in the stomach of STZ-induced diabetic rats. As a result, rescue of ICC networks was associated with a preventing effect on delayed gastric emptying.

As an effective procedure with fewer side effects, EA combined with acupuncture and electrical current stimulation instead of manual manipulations is gradually accepted by Western countries. ST36 is one of the most frequently used points to treat gastrointestinal disorders, with indications including: epigastric pain, nausea, vomiting, poor appetite and abdominal distention. Simultaneously, our previous study has indicated EA at ST36 could increase the colonic propulsive movement but not observed in group with EA at non-acupoints. Delayed gastric emptying is a common character of diabetic patients with gastroparesis and also one of major factors contributing to symptoms of gastroparesis. During the past decades, experiments in conscious rats showed that low frequency EA (10 Hz) significantly accelerated gastric emptying [3], [24], [25]. Additionally, 100 Hz EA was effective to cause the release of dinorphin and serotonin to induce analgesia [26]. Although high frequency EA (100 Hz) was also reported to promote esophageal and distal colon motility in animals [7], [8], the effects of 100 Hz EA on gastric motility have scarcely investigated. Thus, parameters (10 and 100 Hz) of EA were selected according to preliminary experiments that indicated stimulatory effect of gastrointestinal motility. Our results demonstrated that both low frequency EA (10 Hz) and high frequency EA (100 Hz) at ST36 significantly improved delayed gastric emptying in diabetic rats, and the result also revealed that the effect of low frequency EA was more dramatic than that of high frequency EA.

Numerous studies have demonstrated that ICC plays an important role in the regulation of gastric peristalsis, a major determinant of gastric emptying [27], [28]. As ICC exists in different layers of gastric antrum in the form of networks *in vivo*, alterations of ICC networks are valuably to be considered. Reduction in number and morphological changes of ICC-MY and ICC-IM in the stomach of diabetic mice were demonstrated in a previous study [14]. Similarly, a marked loss of ICC-IM and ICC-SM in antrum of STZ-induced diabetic rats was observed, but ICC-MY was not significantly affected except for a loss of connections with nerve structures [15]. These differences of findings in these two studies were probably associated with animal species. From a study of 28 patients with gastroparesis, a reduced number of ICC was found in the myenteric plexus by full-thickness antral biopsies [29]. Our results showed that ICC-IM, ICC-MY and ICC-SM in the antrum of STZ-induced diabetic rats were dramatically reduced based on technique of western blotting and immunofluorescence staining, and further the damaged structure of ICC was showed by transmission electron microscopy. Fortunately, it is reported that low frequency EA improves the expression of ICC in colon of slow transit constipation rats [17]. Additionally, our previous study has suggested that both low and high frequency EA restores the expression of ICC in colon of diabetic rats [18]. However, in those studies, they investigate the alteration of ICC as a whole and the effects of different frequency EA on ICC in the stomach have not been elucidated. Thus, the effects of different frequency EA on three subtypes of ICC in the stomach including ICC-IM, ICC-MY and ICC-SM were the focus of the present study. Our results showed that the expression of ICC in the three layers were conspicuously rescued by low and high frequency EA, suggesting that low and high frequency EA renovated networks of ICC-IM, ICC-MY and ICC-SM and further might contribute to a recovered gastric emptying.

It is noteworthy that the balance of ICC networks is mainly determined by apoptosis and proliferation of ICC [11], [12]. Therefore, the possible mechanism on gastric motility and the effects on ICC networks of EA at ST36 were explored through detecting the alterations of proliferation and apoptosis of ICC. Recently, apoptotic cell death is reported to be an ongoing process of ICC networks and the level of apoptosis in healthy colon indicates that these cells must be continually regenerated to maintain intact networks [12]. Besides, results from adult guinea pigs indicated that intestinal ischemia and reperfusion injury led to reduction in number of ICC through apoptosis which may contribute to gastrointestinal motility disorders [30]. Moreover, a recent report showed that EA therapy reduced the apoptotic percentage of substantia nigra cells in the Parkinsonian rats [31]. The present study has clearly articulated that apoptotic ICC detected by TUNEL methods were flood in ICC networks of diabetic rat stomach and speculated that depletion of ICC networks might also lead to gastric motility disorders. But through the intervention of low and high frequency EA stimulation, TUNEL+ nucleus were hardly to find, suggesting apoptosis of ICC were reduced to a low level as normal.

Proliferation is also another main regulator to maintain networks of ICC. Previous results have shown that proliferation was involved in the recovery of ICC once it was lost in adult animals [32], [33]. In addition, it is reported that acupuncture at ST36 increased cell proliferation in dentate gyrus of STZ-induced diabetic rats [34] and EA enhanced cell proliferation and differentiation in young rat brain [19]. Moreover, EA improved the expression of colonic ICC in slow transit constipation rats and diabetic rats [17], [18]. We demonstrated in the present study that c-kit/Ki67 double labeled ICC-IM, ICC-MY and ICC-SM in the LEA and HEA group reached a high level in density for 8 weeks' EA intervention. Additionally, abundant c-kit+/Ki67+ cells in the LEA group revealed low frequency was more effective on increasing proliferation than high frequency. Therefore, proliferated ICC may be involved in the mechanism of EA at ST36 on recovering gastric motility.

In summary, the expression of ICC was significantly decreased and networks of ICC-IM, ICC-MY and ICC-SM were significantly damaged in the distal stomach of diabetic rats, contributing to a delayed gastric emptying and responsible for some of the major symptoms of diabetic gastropathy and gastroparesis. By reducing apoptosis and increasing proliferation of ICC, both low and high frequency EA at ST36 restored the networks of ICC-IM, ICC-MY and ICC-SM, accompanying with an improved gastric emptying. Therefore, further studies are needed to investigate the mechanisms of EA at ST36 on proliferation and apoptosis of ICC and attach more importance for its clinical potential for the treatment of functional gastrointestinal disorders.

## References

[1] Rodrigues ML, Motta ME (2012) Mechanisms and factors associated with gastrointestinal symptoms in patients with diabetes mellitus. J Pediatr (Rio J) 88: 17–24 10.2223/JPED.2153. PubMed: 22344626 22344626

[2] Sfarti C, Trifan A, Hutanasu C, Cojocariu C, Singeap AM, et al (2010) Prevalence of gastroparesis in type 1 diabetes mellitus and its relationship to dyspeptic symptoms. J Gastrointestin Liver Dis 19: 279–284. PubMed: 20922192 20922192

[3] Imai K, Ariga H, Chen C, Mantyh C, Pappas TN, et al (2008) Effects of electroacupuncture on gastric motility and heart rate variability in conscious rats. Auton Neurosci 138: 91–98 10.1016/j.autneu.2007.11.003. PubMed: 18083640 18083640

[4] Yin J, Chen J, Chen JD (2010) Ameliorating effects and mechanisms of electroacupuncture on gastric dysrhythmia, delayed emptying, and impaired accommodation in diabetic rats. Am J Physiol Gastrointest Liver Physiol 298: G563–570 10.1152/ajpgi.00252.2009. PubMed: 20093561 20093561

[5] Wang CP, Kao CH, Chen WK, Lo WY, Hsieh CL (2008) A single-blinded, randomized pilot study evaluating effects of electroacupuncture in diabetic patients with symptoms suggestive of gastroparesis. J Altern Complement Med 14: 833–839 10.1089/acm.2008.0107. PubMed: 18721079 18721079

[6] Xu S, Hou X, Zha H, Gao Z, Zhang Y, et al (2006) Electroacupuncture accelerates solid gastric emptying and improves dyspeptic symptoms in patients with functional dyspepsia. Dig Dis Sci 51: 2154–2159 10.1007/s10620-006-9412-x. PubMed: 17082991 17082991

[7] Luo D, Liu S, Xie X, Hou X (2008) Electroacupuncture at acupoint ST-36 promotes contractility of distal colon via a cholinergic pathway in conscious rats. Dig Dis Sci 53: 689–693 10.1007/s10620-007-9929-7. PubMed: 17768682 17768682

[8] Shuai X, Xie P, Liu J, Xiang Y, Li J, et al (2008) Different effects of electroacupuncture on esophageal motility and serum hormones in cats with esophagitis. Dis Esophagus 21: 170–175 10.1111/j.1442-2050.2007.00757.x. PubMed: 18269654 18269654

[9] Yin J, Chen JD (2008) Roles of interstitial cells of Cajal in regulating gastrointestinal motility: in vitro versus in vivo studies. J Cell Mol Med 12: 1118–1129 10.1111/j.1582-4934.2008.00352.x. PubMed: 18429936 18429936 PMC3865654

[10] Ibba Manneschi L, Pacini S, Corsani L, Bechi P, Faussone-Pellegrini MS (2004) Interstitital cells of Cajal in the human stomach: distribution and relationship with enteric innervation. Histol Histopathol 19:1153–1164. PubMed: 15375758 15375758 10.14670/HH-19.1153

[11] Farrugia G (2008) Interstitial cells of Cajal in health and disease. Neurogastroenterol Motil 20 Suppl 1 54–63 10.1111/j.1365-2982.2008.01109.x. PubMed: 18402642 18402642

[12] Gibbons SJ, De Giorgio R, Pellegrini MS, Garrity-Park MM, Miller SM, et al (2009) Apoptotic cell death of human interstitial cells of Cajal. Neurogastroenterol Motil 21: 85–93 10.1111/j.1365-2982.2008.01185.x. PubMed: 18798796 18798796 PMC2627790

[13] Forster J, Damjanov I, Lin Z, Sarosiek I, Wetzel P, et al (2005) Absence of the interstitial cells of Cajal in patients with gastroparesis and correlation with clinical findings. J Gastrointest Surg 9: 102–108 10.1016/j.gassur.2004.10.001. PubMed: 15623450 15623450

[14] Ordög T, Takayama I, Cheung WK, Ward SM, Sanders KM (2000) Remodeling of networks of interstitial cells of Cajal in a murine model of diabetic gastroparesis. Diabetes 49: 1731–1739. PubMed: 11016458 11016458 10.2337/diabetes.49.10.1731

[15] Wang XY, Huizinga JD, Diamond J, Liu LW (2009) Loss of intramuscular and submuscular interstitial cells of Cajal and associated enteric nerves is related to decreased gastric emptying in streptozotocin-induced diabetes. Neurogastroenterol Motil 21: 1095–e92 10.1111/j.1365-2982.2009.01336.x. PubMed: 19566589 19566589

[16] Deng JJ, Liu XR, Yuan Q (2011) Effect of acupuncture on gastrointestinal stem cell factor/kit system after colocolic anastomosis in rats. Zhen Ci Yan Jiu 36: 176–180. PubMed: 21793381 21793381

[17] Sun JH, Guo H, Chen L, Wu XL, Li H, et al (2011) Effect of electroacupuncture at "Tianshu"(ST 25) on colonic smooth muscle structure and interstitial cells of cajal in slow transit constipation rats. Zhen Ci Yan Jiu 36: 171–175. PubMed: 21793380 21793380

[18] Juanjuan Xu, Yan Chen, Shi Liu, Xiaohua Hou (2012) Electroacupuncture at Zusanli (ST-36) restores impaired interstitial cells of Cajal and regulates stem cell factor pathway in the colon of diabetic rats. J Evid Based Complement Alternat Med 17: 117–125.

[19] Gao J, Wang S, Wang X, Zhu C (2011) Electroacupuncture enhances cell proliferation and neuronal differentiation in young rat brains. Neurol Sci 32: 369–374 10.1007/s10072-010-0402-6. PubMed: 20852908 20852908

[20] Zhao JX, Tian YX, Xiao HL, Hu MX, Chen WR (2011) Effects of electroacupuncture on hippocampal and cortical apoptosis in a mouse model of cerebral ischemia-reperfusion injury. J Tradit Chin Med 31: 349–355 10.1016/S0254-6272(12)60017-X. PubMed: 22462244 22462244

[21] Di Marzo V, Capasso R, Matias I, Aviello G, Petrosino S, et al (2008) The role of endocannabinoids in the regulation of gastric emptying: alterations in mice fed a high-fat diet. Br J Pharmacol 153: 1272–1280 10.1038/sj.bjp.0707682. PubMed: 18223666 18223666 PMC2275439

[22] Komuro T, Zhou DS (1996) Anti-c-kit protein immunoreactive cells corresponding to the interstitial cells of Cajal in the guinea-pig small intestine. J Auton Nerv Syst 61: 169–174 10.1016/S0165-1838(96)00078-1. PubMed: 8946337 8946337

[23] Tharayil VS, Wouters MM, Stanich JE, Roeder JL, Lei S, et al (2010) Lack of serotonin 5-HT2B receptor alters proliferation and network volume of interstitial cells of Cajal in vivo. Neurogastroenterol Motil 22: 110–e109. – 10.1111/j.1365-2982.2009.01435.x. PubMed: 19941613 PMC2852486 19941613

[24] Iwa M, Nakade Y, Pappas TN, Takahashi T (2006) Electroacupuncture elicits dual effects: stimulation of delayed gastric emptying and inhibition of accelerated colonic transit induced by restraint stress in rats. Dig Dis Sci 51: 1493–500 10.1007/s10620-006-9083-7. PubMed: 16868821 16868821

[25] Ouyang H, Yin J, Wang Z, Pasricha PJ, Chen JD (2002) Electroacupuncture accelerates gastric emptying in association with changes in vagal activity. Am J Physiol Gastrointest Liver Physiol 282: G390–396. PubMed: 11804862 11804862 10.1152/ajpgi.00272.2001

[26] Silva JR, Silva ML, Prado WA (2011) Analgesia induced by 2- or 100-Hz electroacupuncture in the rat tail-flick test depends on the activation of different descending pain inhibitory mechanisms. J Pain 12: 51–60 10.1016/j.jpain.2010.04.008. PubMed: 20554480 20554480

[27] Hirst GD, Edwards FR (2006) Electrical events underlying organized myogenic contractions of the guinea pig stomach. J Physiol 576: 659–665 10.1113/jphysiol.2006.116491. PubMed: 16873400 16873400 PMC1890413

[28] Vittal H, Farrugia G, Gomez G, Pasricha PJ (2007) Mechanisms of disease: the pathological basis of gastroparesis—a review of experimental and clinical studies. Nat Clin Pract Gastroenterol Hepatol 4: 336–346 10.1038/ncpgasthep0838. PubMed: 17541447 17541447

[29] Harberson J, Thomas RM, Harbison SP, Parkman HP (2010) Gastric neuromuscular pathology in gastroparesis: analysis of full-thickness antral biopsies. Dig Dis Sci 55: 359–370 10.1007/s10620-009-1071-2. PubMed: 19997975 19997975

[30] Mei F, Guo S, He YT, Zhu J, Zhou DS, et al (2009) Apoptosis of interstitial cells of Cajal, smooth muscle cells, and enteric neurons induced by intestinal ischemia and reperfusion injury in adult guinea pigs. Virchows Arch 454: 401–409 10.1007/s00428-009-0739-5. PubMed: 19214565 19214565

[31] Wang YC, Cheng YH, Ma J, Gan SY, Wang SJ, et al (2010) Effect of electroacupuncture on morphological changes and apoptosis of substantia nigra cells in Parkinson's disease rats. Zhen Ci Yan Jiu 35: 415–421. PubMed: 21375014 21375014

[32] Mei F, Han J, Huang Y, Jiang ZY, Xiong CJ, et al (2009) Plasticity of interstitial cells of cajal: a study in the small intestine of adult Guinea pigs. Anat Rec (Hoboken) 292: 985–993 10.1002/ar.20928. PubMed: 19548308 19548308

[33] Mei F, Yu B, Ma H, Zhang HJ, Zhou DS (2006) Interstitial cells of Cajal could regenerate and restore their normal distribution after disrupted by intestinal transection and anastomosis in the adult guinea pigs. Virchows Arch 449: 348–357 10.1007/s00428-006-0258-6. PubMed: 16912883 16912883

[34] Kim EH, Jang MH, Shin MC, Lim BV, Kim HB, et al (2002) Acupuncture increases cell proliferation and neuropeptide Y expression in dentate gyrus of streptozotocin-induced diabetic rats. Neurosci Lett 327: 33–36 10.1016/S0304-3940(02)00372-5. PubMed: 12098494 12098494

